# Closed reduction and percutaneous pinning for treatment of unstable lateral condyle fractures of the humerus in children

**DOI:** 10.3389/fped.2023.1223615

**Published:** 2023-08-23

**Authors:** Fei Qiao, Xiaohong Guan, Fei Jiang, Ping Lv

**Affiliations:** ^1^ Department of Pediatric Orthopaedic, Dalian Women and Children's Medical Group, Dalian, China; ^2^ Department of Anesthesia, Dalian Women and Children's Medical Group, Dalian, China; ^3^ Department of Otorhinolaryngology, Dalian Women and Children's Medical Group, Dalian, China

**Keywords:** fractures, children, closed reduction, Kirschner wire, pinning

## Abstract

**Objective:**

In the past, obviously displaced lateral condyle fractures of the humerus in children were treated satisfactorily with open reduction and internal fixation (ORIF). However, in recent years, more studies have mentioned closed reduction and percutaneous pinning (CRPP) of these fractures.

**Methods:**

In this retrospective investigation, the radiographic and clinical results of patients with these fractures that were initially managed with CRPP were newly classified. We classified these fractures into three groups according to the degree and pattern of fracture displacement as identified on four radiographic images. In Type I, the fracture is unstable and displacement is ≥2 mm; In Type II degree I, the fracture is unstable and displacement is >2 mm, with single rotation of fragment; In Type II degree II, the fracture is unstable and displacement is >2 mm, with single rotation of fragment, with rotation of fragment and antero-proximal displacement; In Type III, the fracture is unstable and displacement is >2 mm, with posterior dislocation of elbow joint. We also designed an algorithm for closed reduction of these fractures according to this new classification.

**Results:**

We retrospectively analyzed the radiographic and clinical results of 37 unstable fractures (in 22 boys and 15 girls) that were treated with closed reduction. Twenty-one of 25 (84.0%) type I fractures, which could have been reduced to within 2 mm of residual displacement, were treated with closed reduction and pinning with 2 or 3 Kirschner wires (K wires). Three of 5 (60.0%) type II degree I, 3 of 4 (75.0%) type II degree II, and 3 of 3 (100%) type III fractures were treated with CRPP. In 4 of 25 (16.0%) type I, 2 of 5 (40.0%) type II degree I and 1 of 4 (25.0%) type II degree II fractures, closed reduction failed, so ORIF was implemented. There were no complications, such as nonunion, osteonecrosis of the capitellum, superficial or deep infection, malunion, cubitus varus or valgus, or early physeal arrest.

**Conclusion:**

Although the management of type III fractures may not be more difficult than type II fractures with a rotated fracture fragment, as elbow dislocations are usually easy reducible. This retrospective study showed that type III fractures should not be ignored as a lateral condyle fracture that can be cured with CRPP and that lateral humeral condyle fractures with obvious displacement and rotation can be initially treated with CRPP to achieve satisfactory recovery of the elbow. Kirschner wire (K wire) fixation is recommended to avoid reoperation or anesthesia for hardware removal.

## Introduction

Lateral condyle fractures are the second most common injuries of the elbow in pediatric patients aged 6–10 years old, accounting for 5%–20% of childhood injuries; moreover, approximately 60% of these fractures have at least 2 mm of displacement and require aggressive treatment (1–4). Various classification systems for these fractures have been proposed, including those proposed by Jacob, Lagrange, Rigault, Milch, and Weiss, but few have been sustained (2, 3, 5, 6). Although the Milch classification is used to classify fractures, it does not guide treatment nor predict progression (5). In its early development, the Jacob classification determined whether aggressive treatment was needed. According to the Jacob classification, a Type I fracture is non-displaced, Type II is displacement by 2 mm but with no malrotation, and Type III is displacement with malrotation (2). Jacob Type I fractures are routinely treated without surgery, whereas Jacob Type II and III fractures require aggressive treatment (7, 8).

Open reduction and internal fixation (ORIF) is the main treatment for Jacob Type II and III fractures because it prevents complications caused by inaccurate reduction (2, 4, 9–16). Postoperative complications of open reduction remain among the primary challenges, with an incidence ranging from 0% to 32% (8, 17). In a few recent studies, the Song classification has been proven to be dependable and effective in guiding the management of severe fractures. These articles have shown that closed reduction and percutaneous pinning (CRPP) can effectively treat severe lateral condyle fractures, even Jacob Type III or Song Stage V fractures, while avoiding causing complications and the need for repeat incisions (18–22). The objective of this study was to assess the radiographic and clinical outcomes of patients with these types of severe fractures that were initially cured with CRPP by using a new classification system.

## Patients and methods

**Figure 1:**
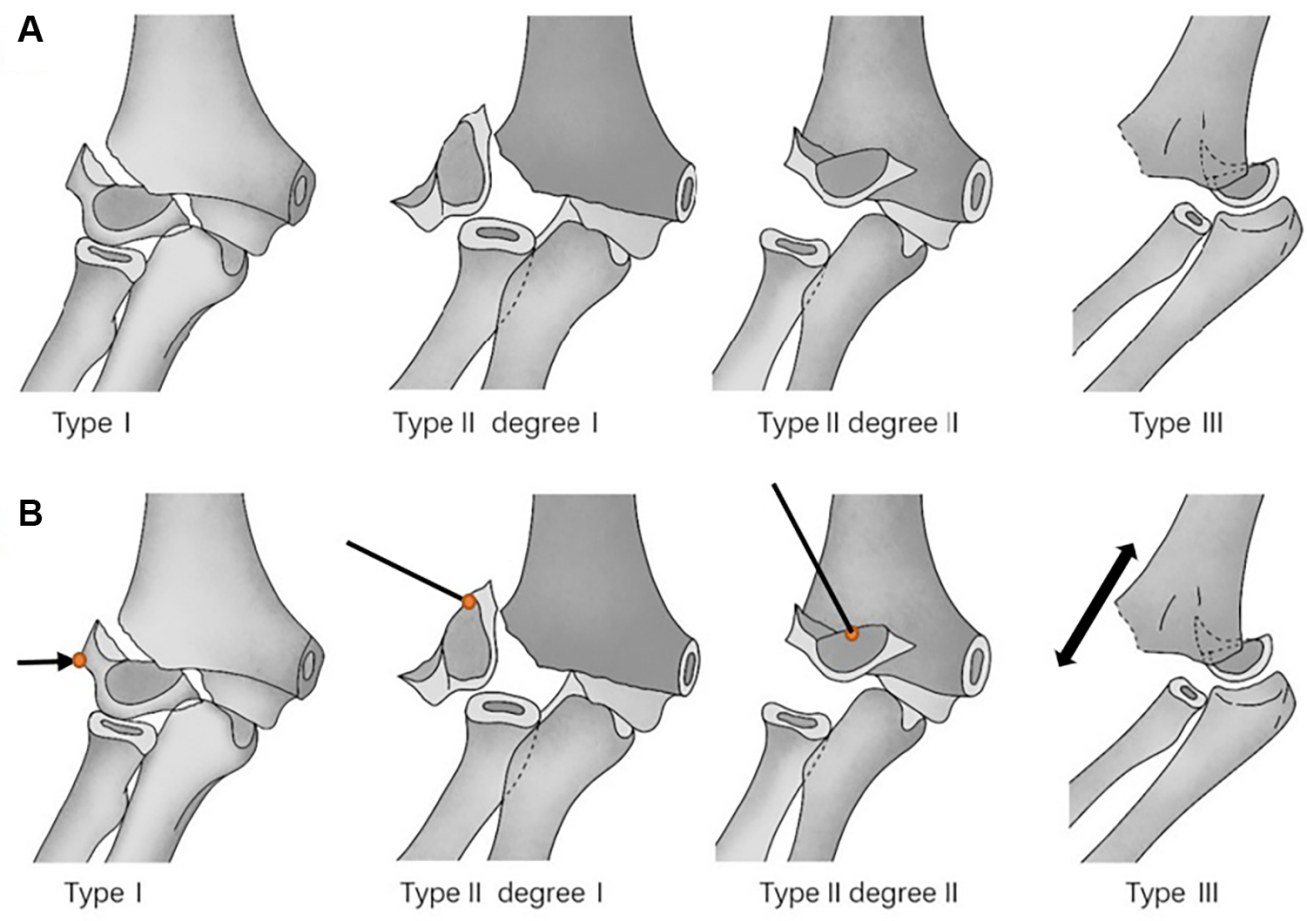
(**A**) Illustrations depicting the classification of displacement more than 2.0 mm of fractures of the lateral condyle of the humerus in children. In Type I, the fracture is unstable and displacement is ≥2 mm. In Type II degree I, the fracture is unstable and displacement is >2 mm, with single rotation of fragment. In Type II degree II, the fracture is unstable and displacement is >2 mm, with single rotation of fragment, with rotation of fragment and antero-proximal displacement. In Type III, the fracture is unstable and displacement is >2 mm, with posterior dislocation of elbow joint. (**B**) A drawing showing schematically the surgical reduction maneuvers in the different types of fracture. In Type I, the surgeon applied gradual pressure on the distal fracture fragments laterally and posteriorly; in Type II degree I, a joystick directly pushing the top of the K-wire on the superior edge of the rotated distal fragment; in Type II degree II, a joystick pushing the top of the K-wire on the medial and superior edges of the distal fragment; in Type III, the dislocated elbow should be reduced first by applying direct traction of the injured arm.

From July 2018 to June 2019, a series of 48 patients with obviously displaced lateral humeral condyle fractures in which the fragment displaced more than 2 mm were treated at our hospital. One surgeon insisted on performing open reduction, and eight patients underwent ORIF without CRPP. Two patients with incomplete radiographic and clinical records and who were not followed up for more than 1 year were excluded, and one patient with osteogenesis imperfecta was also excluded. Thirty-seven out of 40 patients who were initially treated with CRPP were retrospectively evaluated. All 37 patients were operated on by a single senior pediatric orthopedist in our ward. Our classification was designed to classify these fractures (Table 1 and Figures 1A,B). The pattern and extent of fracture displacement were diagnosed three times by two experienced orthopedic surgeons using a hospital information systerm (HIS) (Neusoft Corp., PACS 5.5, DL, China) on the lateral metaphyseal cortex between the humerus and fracture fragment on the anteroposterior (AP) and internal oblique radiographic images (23). The greatest displacement of the posterior cortex on four images was identified as the extent of fracture displacement (21).

**Table 1:** Classifications and reduction according to fracture displacement and pattern.

Type	Degree	Amount of displacement	Fracture pattern	Radiograph views used as basis	Stability	Reduction steps
I		≥2 mm	Without rotation of fragment	Any of four views (especially internal oblique view)	Unstable	Manual reduction
II	Degree I	>2 mm	With single rotation of fragment	Any of four views	Unstable	Step:1 leverage reduction from lateral side
						Step:2 manual reduction
	Degree II	>2 mm	With rotation of fragment and antero-proximal displacement	Any of four views (especially lateral view)	Unstable	Step:1 leverage reduction from anterolateral side
						Step:2 manual reduction
III		>2 mm	Fractures with posterior dislocation of elbow joint	Any of four views	Unstable	Step:1 manual reduction of elbow dislocation
						Step:2 manual or leverage reduction of fracture

All the guardians of the patients signed written informed consent forms, and the research was approved by the Institutional Ethical Review Board of our hospital (approval number DLET-KY-2022-38). All methods were performed in accordance with the relevant guidelines and regulations.

The results were evaluated in terms of the range of elbow motion, the carrying angle, and the incidence of other complications, including delayed union, infection, osteonecrosis, or cubitus varus or valgus. The elbow was evaluated on the basis of Hardacre et al. (15) (Table 2). The modified Clavien‒Dindo classification was used to classify the complications (24).

**Table 2:** Evaluation of results by Hardacre et al.

	Range of motion	Carrying angle	Symptom
Excellent	No limitation	No alteration	No symptoms
Good	Functional range of motion (lacking no more 15° of complete extension)	Inconspicuous	No arthritic, neurologic symptoms
Poor	Disabling loss of function	Conspicuous alteration	Arthritic symptom, ulnar neuritis, Roentgen findings of non-union, avascular necrosis

### Surgical technique

The patients lied in the supine position on the operating table with the injured elbow abducted. After the general anesthesia was induced and the skin was disinfected, the injured elbow draped and underwent manual treatment.

**Figure 2:**
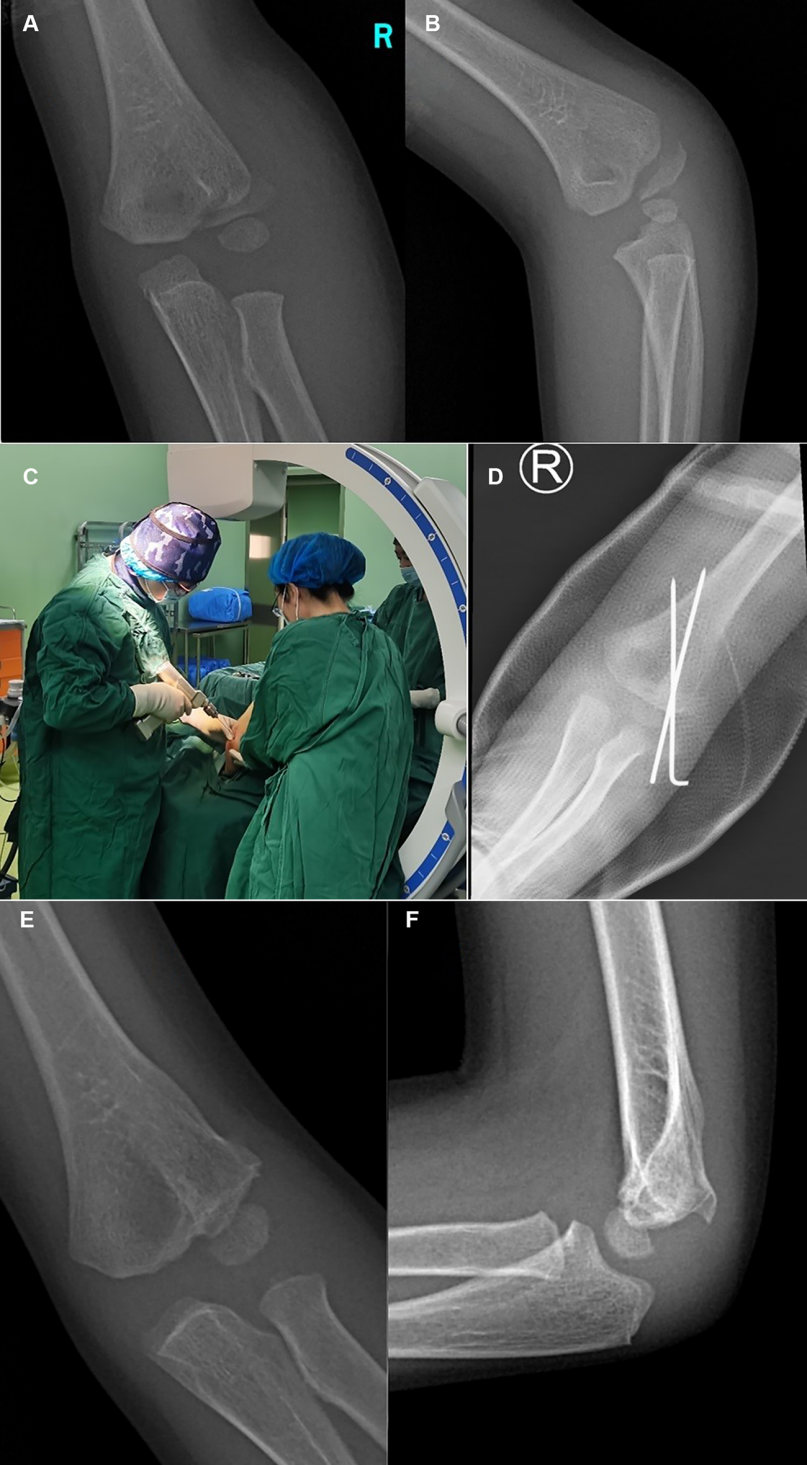
Typical x-ray of Type I fractures. Anteroposterior (AP) (**A**) radiographic image of the injured elbow, showing a Type I fracture with displacement of >2 mm. Internal oblique (**B**) radiographic image of the injured elbow, displaying a Type I fracture with even more obvious fracture fragment displacement. The position (**C**) of the assisting surgeon and operating surgeon during manipulation. (**D**) AP radiograph of the right elbow that was treated with two different 1.6 mm K-wires in CRPP. Typical X-ray of type I. AP (**E**) and lateral (**F**) radiographic images showing fracture union at 10 months postoperatively.

For Type I fractures (Figures 2A,B), taking the right injured arm as an example, the assisting surgeon stood on the right side of the operating surgeon and flexed the injured elbow to approximately 90°. His left hand fixed the upper arm, supinated the forearm, and applied a posterior valgus force to the affected elbow joint. Using the thumb, the surgeon applied gradual pressure on the distal fracture fragments laterally and posteriorly (Figures 2C,D). This method can be performed two to three times to help achieve closed reduction within 2 mm in most cases, as judged by the C-arm intensifier.

**Figure 3:**
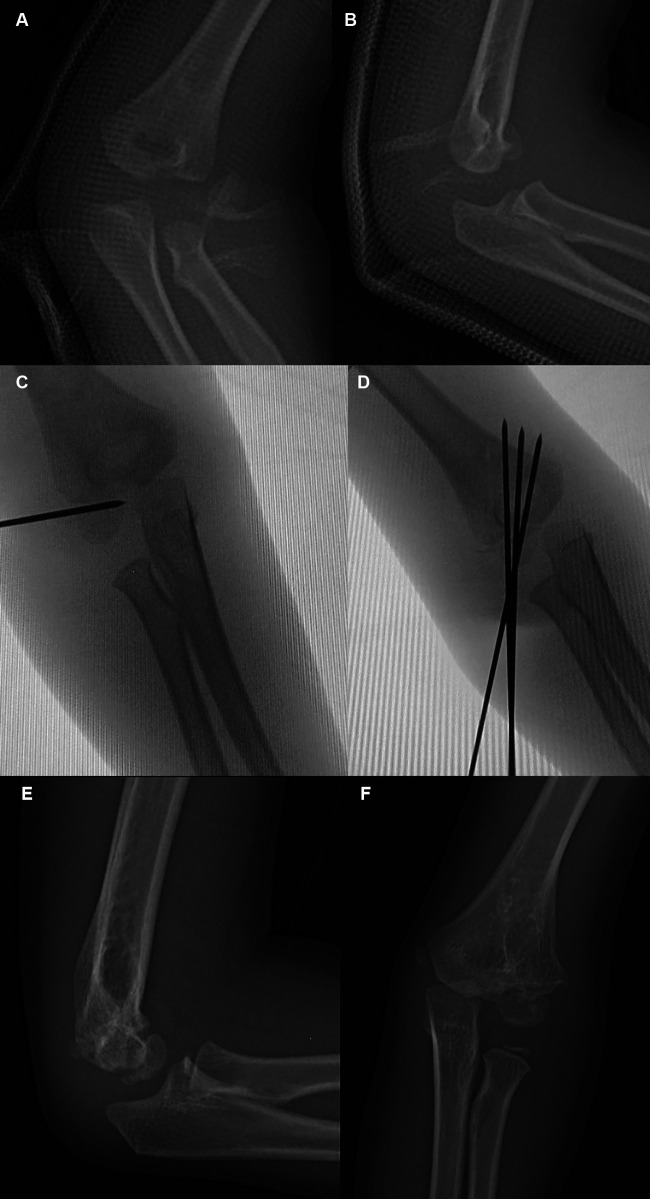
Typical x-ray of Type II degree I fractures. Anteroposterior (AP) (**A**) and lateral radiograph (**B**) showing a severe fracture with a single rotation of the distal fragment. The fracture is classified as a Type II degree I (unstable) fracture. A C-arm radiograph (**C**) showing leverage reduction with a joystick from the lateral side. (**D**) AP image showing almost satisfactory reduction of the fracture. Typical X-ray of Type II and Degree I. Lateral (**E**) and AP (**F**) radiographic images at 7 months postoperatively.

**Figure 4:**
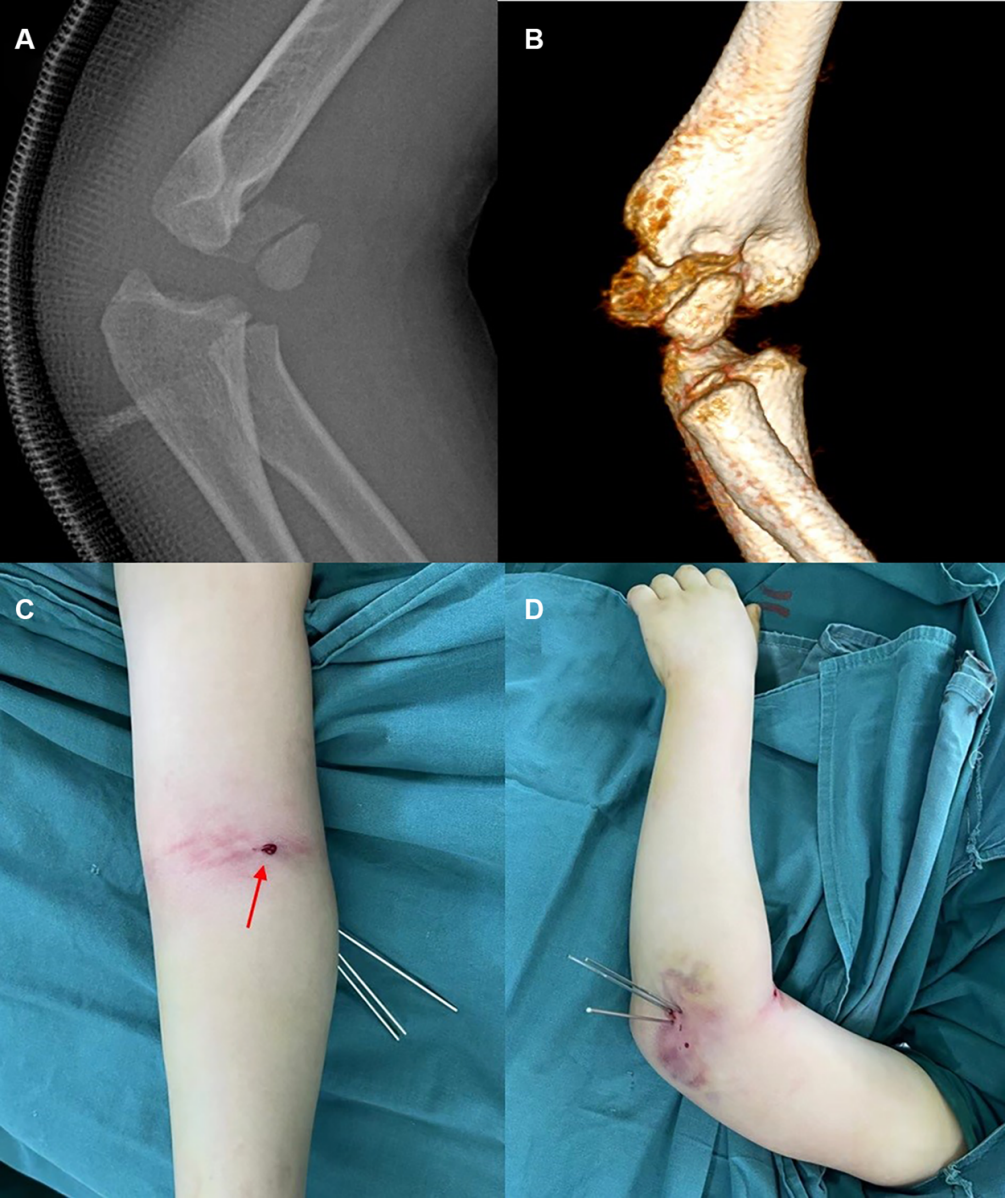
Typical x-ray of Type II degree II fractures. Lateral (**A**) radiograph and 3D CT (**B**) radiograph showing a severe fracture with rotation and antero-proximal displacement of the distal fragment. The fracture is classified as a Type II degree II (unstable) fracture. Aspect (**C**) of leverage reduction from the anterolateral side (red arrow). Aspect (**D**) of the elbow after K-wire fixation.

**Figure 5:**
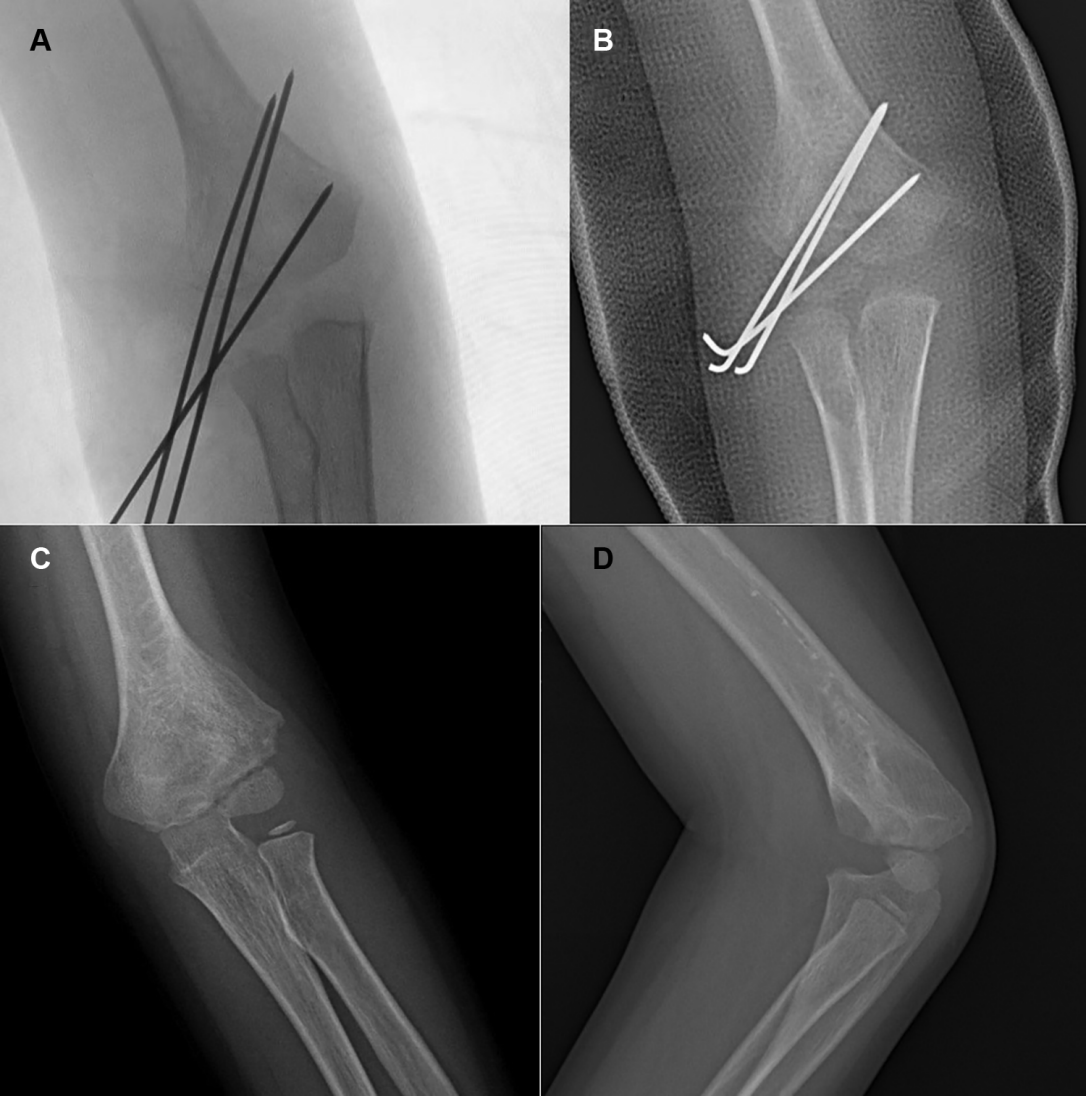
Typical x-ray of Type II degree II fractures. AP (**A**) images showing satisfactory reduction of the fracture. AP (**B**) image of the fracture before removal of the cast and K-wires at 6 weeks postoperatively. AP (**C**) and lateral (**D**) radiographic images at 7 months postoperatively.

For Type II, degree I fractures (Figures 3A,B), the elbow joint should be flexed to approximately 90°, the lateral skin of the elbow should be punctured, and the percutaneous leverage technique should be performed by inserting a Kirschner wire (K-wire), with a diameter of 1.5 or 2.0 mm, into the space between the fragments to act as a joystick and directly pushing the top of the K-wire on the superior edge of the rotated distal fragment to reduce the horizontal rotation and medial edge and decrease the single rotation on the coronal plane (Figures 3C,D). For degree II fractures (Figures 4A,B), the elbow joint should be flexed to approximately 90°, the anterolateral skin of the elbow should be punctured (Figures 4C,D), and the percutaneous leverage technique should be performed by inserting a K-wire, with a diameter of 1.5 or 2.0 mm, into the space between the fragments to act as a joystick, and directly pushing the top of the K-wire on the medial and superior edges of the distal fragment to reduce the proximal displacement and rotation simultaneously. After repositioning, the fracture should be reduced to Type I, and then the above reduction method for Type I should be applied (Figures 5A,B).

**Figure 6:**
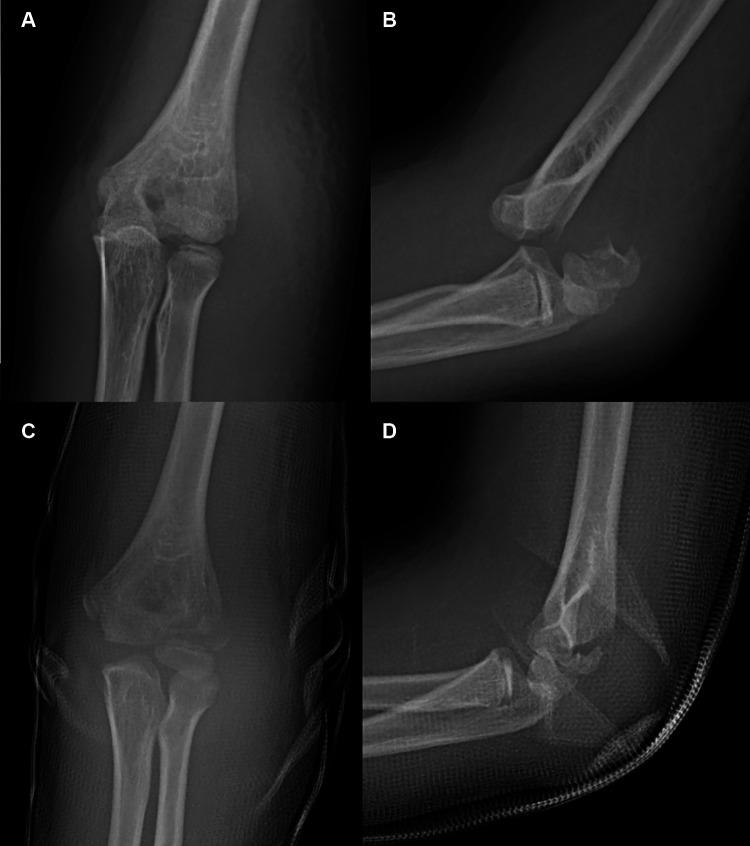
Typical x-ray of Type III fractures. AP (**A**) radiograph and lateral (**B**) radiograph showing a fracture with dislocation of the elbow. The fracture is classified as Type III. AP (**C**) radiograph and lateral (**D**) radiograph showing a Type III fracture reduced to Type I after reduction of the dislocation.

**Figure 7:**
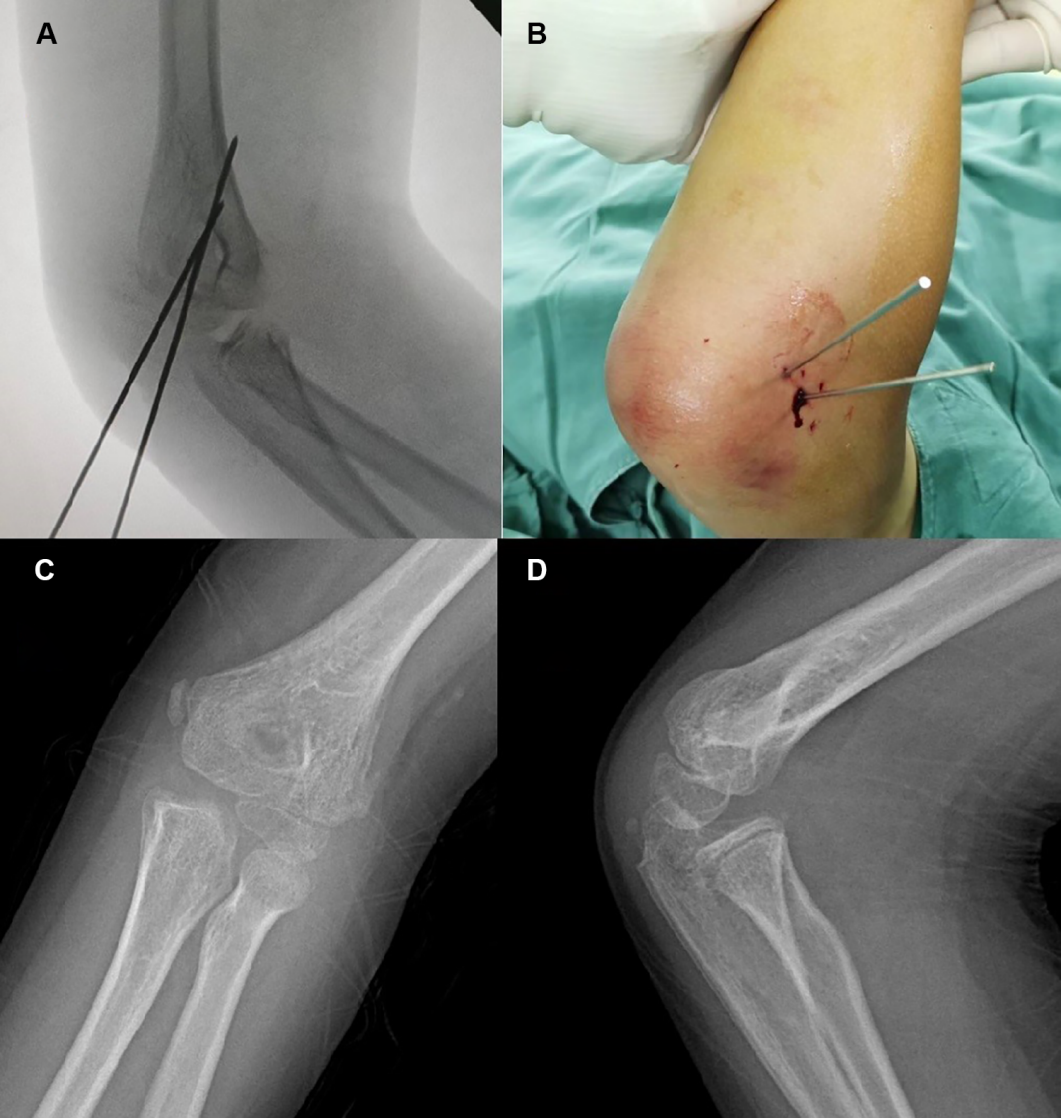
Typical x-ray of Type III fractures. Internal oblique (**A**) image of the fracture showed satisfactory reduction. Aspect (**B**) of the elbow after K-wire fixation. AP (**C**) and lateral (**D**) radiographs 5 months postoperatively.

For Type III fractures (Figures 6A,B), the dislocated elbow should be reduced successfully by applying direct traction of the injured arm; however, the lateral condyle fracture will remain displaced, and the joints will remain unstable (Figures 6C,D). The three fractures should be reduced to Type I after the dislocation is reduced, and then the above reduction method for Type I should be applied to achieve no more than 2 mm displacement of the fracture as judged by the C-arm intensifier (Figures 7A,B).

Percutaneous pinning fixation using two or three divergent 1.6 mm smooth wires should be performed to fix the reduction. The stability of the elbow should be assessed after fixation. If closed reduction fails, ORIF should be performed. After the long-arm plaster cast immobilizes the treated area for 4–7 weeks, the K-wires should be removed (average: 4.7 weeks, range: 4–7 weeks) in the outpatient department without general anesthesia (Figures 2E,F, 3E,F, 5C,D, 7C,D).

SPSS software, version 22 (IBM Corp., Armonk, NY, USA), was utilized for statistical analysis. A kappa value of ≥0.75 indicated a highly significant agreement and was used to assess the interobserver and intraobserver reliability. The paired-samples *t*-test was utilized to assess the results between preoperative and postoperative displacement. The Mann‒Whitney *U* test for independent samples was used for non-normally distributed data. A *p*-value of <0.05 was considered significant.

## Results

In total, 37 patients were included; 22 boys and 15 girls whose ages ranged from 1 year 11 months to 12 years 3 months (mean age: 6 years 3 months). The right elbow was affected in 13 of the patients, and the left elbow was involved in 24 of the patients. Twenty-one of 25 (84.0%) Type I fractures, which could have been reduced to within 2 mm, were treated with closed reduction and pinning using two or three K-wires. Three of five (60.0%) Type II degree I, three of four (75.0%) Type II degree II, and three of three (100.0%) Type III fractures were treated with CRPP. The kappa value (range, 0.798–0.932) indicated a highly significant and reliable interobserver and intraobserver agreement. All the children underwent surgery within 3 days after sustaining trauma. The mean follow-up period was 1 year 6 months (range: 1 year 2 months to 2 years 11 months). Seven of 37 fractures were managed with ORIF. The average initial displacement of the fragment was 8.6 mm (range, 3.9–15.8), and the postoperative residual displacement was 0.6 mm (range, 0.4–1.1 mm) on AP and internal oblique radiographic images. The average operative time of CRPP was 30 min (range, 18–45 min). The mean time for casting and hardware removal was 4.7 weeks (range, 4–7 weeks) in the outpatient department. All fractures were united following open and closed reductions. There was no superficial or deep infection, osteonecrosis of the trochlea or capitellum, malunion, early physeal arrest, or cubitus varus or valgus. Clinical outcomes were assessed using the standard described in Hardacre et al. (Table 2) and were considered excellent in 26 of 30 (86.7%) patients, good in four patients, and poor in none of the patients. The function of all the elbows were satisfactory, except for three cases ranging from 5° to 10°, and the results were good.

## Discussion

This article highlights the possibility of reducing severely displaced and rotated lateral condyle fractures associated with elbow dislocation in children. Careful preoperative estimation of these fractures is critical, as accurate preoperative evaluation by our new classification system allows for appropriate management. This study is the first study in which the authors classified LC fractures in combination with posterior elbow dislocation as one stage to indicate their suitability for management with CRPP. A total of 81.1% (30 of 37) of obviously displaced fractures could be treated with CRPP. Clinical results were excellent in 86.7% (26/30) of patients, good in 13.3% (4/30) of patients, and poor in no patients. CRPP is generally recommended for these fractures to obtain sufficient fracture reduction and good results (18–22).

Various classification systems, including those created by Rigault, Jacob, Lagrange, Weiss, and Song, have been proposed for these fractures (2, 3, 6, 25). Ramo et al. (19) and Xie et al. (22) found that the Song classification had a satisfactory reliability and predictive ability while effectively guiding treatment. However, lateral condyle fracture with elbow dislocation is not mentioned as a type of fracture in any of these classifications. Sharma et al. (26) suggested that the complex elbow anatomy, with characteristic appearances at different ages before skeletal immaturity, could contribute to a misdiagnosis. Wiekrykas et al. (27) reported that a pediatric lateral condyle fracture with elbow dislocation was treated with ORIF using two 4.0 cannulated screws, and the elbow function of the patient recovered well. However, a secondary surgery was needed to remove the hardware. Sharma et al. (26) reported a series of 12 lateral condylar fractures associated with elbow dislocation in children. Their management included elbow dislocation that was first treated by manual reduction, followed by ORIF (K-wire, *n* = 3; screw, *n* = 9); three of 12 had unsatisfactory outcomes with loss of extension at the last follow-up. In our group, all the three lateral condylar fractures associated with elbow dislocation, classified as Type III and Milch II, were successfully treated with manual reduction of the elbow joint and distal fracture fragment and percutaneous pinning. The results of all the three cases were excellent. We believe that these fractures should be carefully evaluated preoperatively to avoid the need for open reduction and should be seriously considered as an additional type of lateral condyle fracture.

We agree with Song, Xie, Mintzer, and Ramo et al. (18–22) that severe lateral condyle fractures can be cured with CRPP, and approximately 75% of completely displaced and rotated fractures were reduced to within 2 mm in previous reports. Our article showed similar results when CRPP was used to manage lateral condylar humerus fractures (LCHFs). Approximately 81.1% (30 of 37) (21 of 25 Type I fractures, three of five cases of Type II degree I, three of four cases of Type II degree II, and three of three cases of Type III) of our cohort were treated with CRPP. The last follow-up showed no major complications or dysfunction except for loss of elbow extension (5°–10°) without symptoms in most cases. CRPP could be performed in a mean of 30 min (range, 18–45 min). Compared with the time of open reduction and pinning for seven of the 37 fractures, the time for pinning required an average of 45 min (range, 34–56 min). CRPP had a shorter operative time (*p* < 0.05).

Weiss et al. reported the largest cohort of patients with severe lateral condyle fractures cured with CRPP to date, which was composed of 65 patients. They advised that patients with fractures displaced between 2 and 4 mm undergo manual reduction and K-wire fixation; nevertheless, patients with greater displacement should undergo open reduction (6). Song et al. (21) and Xie et al. (22) expanded the indications for CRPP to include rotated fractures and obtained satisfactory results without causing minor or major complications. Within our cohort, eight patients were treated with open reduction because one surgeon who insisted on performing CRPP could not directly visualize the joint surface. A total of 30 (out of 37) patients underwent initial CRPP because it was less invasive and reduces the incidence of complications. All 37 fractures were treated by a single senior pediatric orthopedist in our ward. A comparison between the two groups showed no significant differences in the clinical and radiological outcomes, and CRPP was beneficial with shorter surgery time and no incision needed. Therefore, our results corroborated those reported by Song et al. (21) and Xie et al. (22), who concluded that CRPP might be the preferred treatment for a new classification of lateral condyle fractures. Our findings suggest that these fractures are possibly suitable for treatment with CRPP.

By reviewing the published articles, we found that screws and K-wires could be used to fix these fractures. Schlitz et al. (28) insisted that compared with K-wires, screw fixation increased stability. Li and Xu (29) showed that screws can reduce the appearance of lateral prominence and promote the recovery of elbow function, but a second surgery is needed for hardware removal. To the best of our knowledge, both cannulated screw implantation and K-wire fixation are effective in the treatment of these fractures. Smooth wires can pass through the ossified nucleus of the capitulum, but local skin care must be advised. Ultimately, we chose K-wires for internal fixation for our patients, and local skin care was performed every fortnight to reduce irritation of the shin skin. Two patients experienced skin irritation, which resolved after the hardware was removed.

Song et al. (4, 18, 21) recommended a longer learning curve for the proper evaluation of fracture models and the implementation of manual reduction, and precise evaluation of the displacement of fractures and standardized intraoperative identification of the position on both AP and internal oblique radiographs led to the high success rate (75%) of closed reduction and internal fixation (CRIF), and stable fixation of the reduction with divergent K-wires was necessary. CRIF failed in seven of 37 cases, and then ORIF was used for the treatment of these fractures, achieving good and excellent results and no severe complications. The possible reasons explaining the failure of CRIF were as follows: (1) at the early period of our study, a learning period was essential for correctly understanding the fracture patterns and proper conduction of closed reduction; (2) it is difficult to manipulate and maintain the reduction by K-wires of younger children with smaller distal fragments; and (3) for Type II fractures, the difficulty lied in the correction of rotation by 2.0 mm K-wire as a joystick with the elbow joint flexion that had been mentioned above in the “Surgical technique” section.

Our research showed that CRPP had a high success rate (30/37, 81.1%) for the treatment of these fractures. CRPP achieved excellent results in treating these fractures, even Type III fractures. We acknowledge that the cohort of patients was small and the period of follow-up was short, and additional randomized, prospective studies comparing open and closed reduction are necessary to further assess the utility of this method. We believe that the success rate of CRPP was due to the following: (1) precise evaluation of the fracture displacement pattern, which was mainly anterolateral; (2) maintenance of the reduction with two or three percutaneous K-wires; (3) routine intraoperative C-arm confirmation of the reduction on both AP and internal oblique radiographs, as advised by Song et al. (18); and (4) our insistence in using our reduction strategy according to our classification with elbow joint flexion to improve the success rate of closed reduction.

In conclusion, this retrospective study showed that lateral humeral condyle fractures with obvious displacement and rotation could be initially treated with CRPP and that doing so achieved satisfactory recovery of elbow function, and also that Type III fractures, according to our new classification, should be seriously considered as an additional type of lateral condyle fracture that is suitable for treatment with CRPP. K-wire fixation should be advised to avoid reoperation or anesthesia for hardware removal.

## Data Availability

The original contributions presented in the study are included in the article, further inquiries can be directed to the corresponding author.
